# Efficient Mobility Management Signalling in Network Mobility Supported PMIPV6

**DOI:** 10.1155/2015/539394

**Published:** 2015-08-16

**Authors:** Ananthi Jebaseeli Samuelraj, Sundararajan Jayapal

**Affiliations:** ^1^CSE Department, Anna University, Chennai, Tamil Nadu 600025, India; ^2^Pavai College of Technology, Namakkal, Tamil Nadu 637018, India

## Abstract

Proxy Mobile IPV6 (PMIPV6) is a network based mobility management protocol which supports node's mobility without the contribution from the respective mobile node. PMIPV6 is initially designed to support individual node mobility and it should be enhanced to support mobile network movement. NEMO-BSP is an existing protocol to support network mobility (NEMO) in PMIPV6 network. Due to the underlying differences in basic protocols, NEMO-BSP cannot be directly applied to PMIPV6 network. Mobility management signaling and data structures used for individual node's mobility should be modified to support group nodes' mobility management efficiently. Though a lot of research work is in progress to implement mobile network movement in PMIPV6, it is not yet standardized and each suffers with different shortcomings. This research work proposes modifications in NEMO-BSP and PMIPV6 to achieve NEMO support in PMIPV6. It mainly concentrates on optimizing the number and size of mobility signaling exchanged while mobile network or mobile network node changes its access point.

## 1. Introduction

PMIPV6 [1] is initially designed to support single mobile node movement. Many use cases or scenarios [2] necessitate mobility support in PMIPV6 network. The mobility signaling and the data structures used in PMIPV6 are designed for individual mobile node's mobility management. So PMIPV6 needs significant changes to support group nodes' movement. This section describes basic PMIPV6 [1], NEMO-BSP [3], issues arising while NEMO-BSP is directly applied in PMIPV6, and the enhancements proposed in PMIPV6 and NEMO-BSP to support NEMO in PMIPV6.

### 1.1. PMIPV6 Network

PMIPV6 [1] protocol is designed to track mobile node's movement and exchanges mobility signaling on behalf of mobile nodes. PMIPV6 network consists of network elements, namely, Local Mobility Anchor (LMA) and Mobile Access Gateway (MAG). MAG acts as an access point to the mobile nodes in PMIPV6 network and routes packet between LMA and mobile node. LMA is the only central point to communicate with the nodes, which are present inside or outside of PMIPV6 network. One LMA serves many MAGs. The main objective of PMIPV6 is that IP address of mobile node should not be changed, even if the mobile node changes its access point in PMIPV6 network. While a mobile node joins in PMIPV6 network, MAG detects its attachment. Then, mobility signaling Proxy Binding Update (PBU) and Proxy Binding Acknowledgment (PBA) are exchanged between MAG and LMA to allocate mobile node's Mobile Network Prefix (MNP). MAG sends this MNP in Router Advertisement (RA) to mobile node. Upon receiving MNP, mobile node configures its address. Afterwards, if the mobile node changes its point of attachment in PMIPV6 network, same procedure is repeated and LMA allocates the same network prefix to the mobile node. So even if the mobile node changes its point of attachment, mobile node's IP address is not changed and applications running in mobile node are not aware of the mobile node movement.

### 1.2. NEMO-BSP

NEMO-BSP [3] supports mobile network movement in Mobile IPV6 (MIPV6) [4] network. Mobile network consists of a mobile router, Locally Fixed Nodes (LFN), and mobile nodes (MN). Mobile nodes configure their address based on the Home Network Prefix (HNP) advertised by MR and MR acts as their fixed access point. If a mobile network joins in foreign network, it obtains its Care-of-Address (CoA) and informs it to the home agent (HA) in Binding Update (BU), so that future packets destined to mobile router will be routed to the new Care-of-Address (CoA). The Binding Update also includes the list of HNPs which are being used inside mobile network. So the home agent can forward all the packets, which are destined to the mobile nodes in mobile network, through mobile router. Thus, the mobile router does not exhibit its mobile network infrastructure to the foreign network but still supports packets forwarding functionality to the nodes in mobile network, without changing their IP Address.

### 1.3. Issues in NEMO-BSP and PMIPV6 Integration

IP address of the nodes in mobile network is allocated based on the Home Network Prefix (HNP) advertised by MR. But nodes in PMIPV6 network have their IP addresses based on MNP given by LMA. So movement between PMIPV6 network and mobile network brings change in IP address of the mobile node, which violates the basics of PMIPV6 network. Hence, all the nodes, which are present in mobile network, should be registered with LMA. Whenever the mobile network changes its point of attachment, LMA should be updated about the new location of all the nodes in mobile network. This causes a lot of mobility signaling exchanges and hence increases load at LMA. As the basic PMIPV6 is designed for single mobile node movement, it shows performance degrade while handling group nodes' movement.

This paper enhances PMIPV6 and NEMO-BSP in such a way that mobile network movement is handled in PMIPV6 network efficiently, without compromising existing functionalities in the underlying protocols. It mainly concentrates on optimizing the number and size of mobility signaling exchanged while mobile network or mobile network node changes its access point.

Section 2 discusses the existing architectures which support NEMO in PMIPV6 and their shortcomings. The proposed architecture is explained in Section 3.  Section 4 compares the performance analysis of the proposed architecture and the existing architectures.  Section 5 concludes the paper.

## 2. Existing Architectures

There are quite a few architectures available to support network mobility in PMIPV6 network. All the architectures exhibit certain shortcomings and they are discussed below.

In rNEMO [5], mobile router acts as a simple relay station for the nodes in mobile network. It simply forwards the packets which are destined to mobile node. The disadvantage in this approach is that LMA should be updated about the exact location of the mobile network, even if mobile node does inter-/intramobile network movement under the same MAG.

Light-weight network mobility within PMIPV6 [6] provides an architecture which supports network mobility in PMIPV6. It does not attempt to reduce mobility signaling while mobile network or mobile network node changes its access point. It also fails to optimize while the mobile node changes its access point under the same MAG (i.e., movement between mobile networks which are connected to same MAG and movement from MAG to mobile network and vice versa).

In N-PMIPV6 [7] protocol, LMA treats mobile nodes which are connected directly to the MAG and the mobile nodes which are connected to mobile router differently. In Proxy Binding Cache Entry (PBCE), LMA maintains the mobile router information with which mobile node is attached, instead of MAG information. Only the mobile router's PBCE has the MAG information. If mobile network changes its access point, location update is sent only for the mobile router. In PBCE, only one entry which is related to mobile router needs the update. All other mobile node's entries in PBCE are kept intact. This avoids a lot of mobility signaling exchange during mobile network movement. But this architecture needs double lookup and tunneling whenever the mobile nodes receive packet. This incurs high communication cost. Also mobility signaling should be exchanged, even if mobile node undergoes intermobile network movement under the same MAG or attaches to the same MAG with which mobile network is attached. This causes high mobility signaling cost, even if the mobile node roams and attaches to different access points under the same MAG.

RENEMO [8] finds a solution to avoid multiple tunneling. LMA always tunnels the packet to the MAG, irrespective of whether the destination node is directly attached to the MAG or attached via a mobile router. MAG takes responsibility for sending the packets to the proper destination node. It avoids multiple tunneling. Also, LMA maintains information about all the networks which are connected to the MAG. While mobile network changes its access point, mobility signaling is exchanged only for the mobile router. Information about the mobile nodes which are connected to the mobile network and their allocated MNPs are sent by LMA to MAG. This reduces mobility signaling cost whenever the mobile network changes its access point. But this method also necessitates mobility signaling to be exchanged, even if mobile node does intermobile network movement under the same MAG or attaches to the same MAG with which mobile network is attached. So the problem of sending mobility signaling, even if the mobile node roams and attaches to different access point but is present under the same MAG, remains unaddressed. Also in this architecture, MAG avoids verifying authentication of mobile nodes present in mobile network, which is very dangerous.

N-NEMO [9] addresses the above said problem. MAG defers sending location update message to LMA, as long as mobile node roams under the same MAG. But whenever the mobile network changes its access point, Binding Update and Binding Acknowledgment are exchanged for all the mobile nodes, which incurs high mobility signaling cost and processing cost at LMA and MAG, whenever mobile network changes its access point.

Lee et al. [10] and Yan et al. [11] provide architectures to support network mobility in PMIPV6. Mobile router acts as moving MAG. Similar to N-PMIPV6, this architecture also suffers the problem of multiple tunneling. It necessitates mobility signaling to be exchanged even if the mobile node attaches to different point under the same MAG, that is, intermobile network mobility and mobile network to MAG and vice versa.

Points inferred from the above analysis are that (i) if nodes in mobile network are handled as group, then signaling cost is reduced, whenever the mobile network changes its access point. But it needs mobility signaling to be exchanged, whenever the mobile node attaches to different access point under the same MAG. (ii) If group is not maintained, it incurs high mobility signaling cost and processing cost, whenever the mobile network changes its access point.

The proposed architecture takes the advantage of the existing approaches and overcomes the problem associated with them. It adds intelligence to the mobile router of the mobile network. MR tracks the mobile network node (MNN) movement in and out of mobile network. It maintains delta network changes (i.e., new nodes joined and nodes left) of mobile network. While the mobile network changes its access point, it gives the list of nodes present in the mobile network, their MNP, and the delta network changes to the MAG. Now MAG knows the nodes present in mobile network. Then, it sends only the delta network changes to the LMA. LMA can derive the complete mobile network information using the delta network changes received from MAG and Proxy Binding Cache Entry (PBCE) maintained by it. Now LMA and MAG are in sync about the mobile network and can start transmitting the packets to and from the mobile network.

## 3. Proposed Architecture

This section discusses the proposed architecture and the way it handles mobile network registration and movement efficiently.

### 3.1. Initial Attachment of Mobile Network

**Figure 1:**
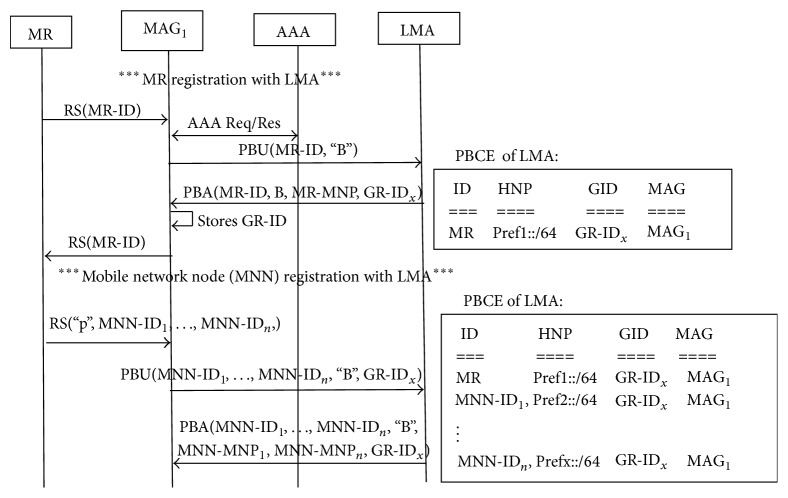
Registration of mobile network with PMIPV6 network.

RENEMO [8] describes the mobile network attachment with PMIPV6 network. Mobile router registers itself with LMA and obtains its Group ID (GR-ID) along with MNP. Then, it registers all the mobile nodes in mobile network with LMA and obtains their MNP. LMA groups all the nodes which belong to the mobile network under the same GR-ID and stores the information in Proxy Binding Cache Entry (PBCE). Registration procedure is shown in Figure 1. MAG verifies the authentication of mobile router and mobile network nodes. Kim and Lee [12] and Lee and Chung [13] have defined architecture for authentication of mobile nodes in PMIPV6 network. As authentication procedure is out of the scope of this paper, it is not described in detail.

LFNs in the mobile network are configured with the Home Network Prefixes (HNPs) belonging to the mobile network. Packets destined to the HNPs of mobile network are always routed to MR. Then, MR forwards those packets to the destination LFNs. As the HNP allocation is permanent and the path to reach the HNP is always via mobile router, the information of the HNP and its corresponding LFNs is not maintained in PBCE.

### 3.2. Mobile Network Tracker List

In the mobile network tracker list, MR maintains the list of nodes which have joined in or left the mobile network. The aim of mobile network tracker list is to capture the delta mobile network difference, after the mobile network is attached to new access point. So MR resets the mobile network tracker list and starts a fresh list, whenever it joins the new MAG. The subsection below explains different scenarios of MNN movement and update of mobile network tracker list in each scenario.

#### 3.2.1. Intra-MAG Movement

In N-NEMO, MAG defers sending PBU to LMA as long as node is roaming under the same MAG. MAG maintains the Proxy Binding Update List (PBUL) to store information about the nodes which are directly connected to or connected via mobile router. PBUL has mobile node ID, its MNP, and the mobile router with which mobile node is attached. If a mobile node changes its access point under the same MAG, (i.e., intermobile network movement under the same MAG and movement between mobile network and MAG), then mobile node's MNP can be read from PBUL itself. PBU is not sent to LMA. This architecture avoids sending PBU for mobile node's intra-MAG movement.

**Figure 2:**
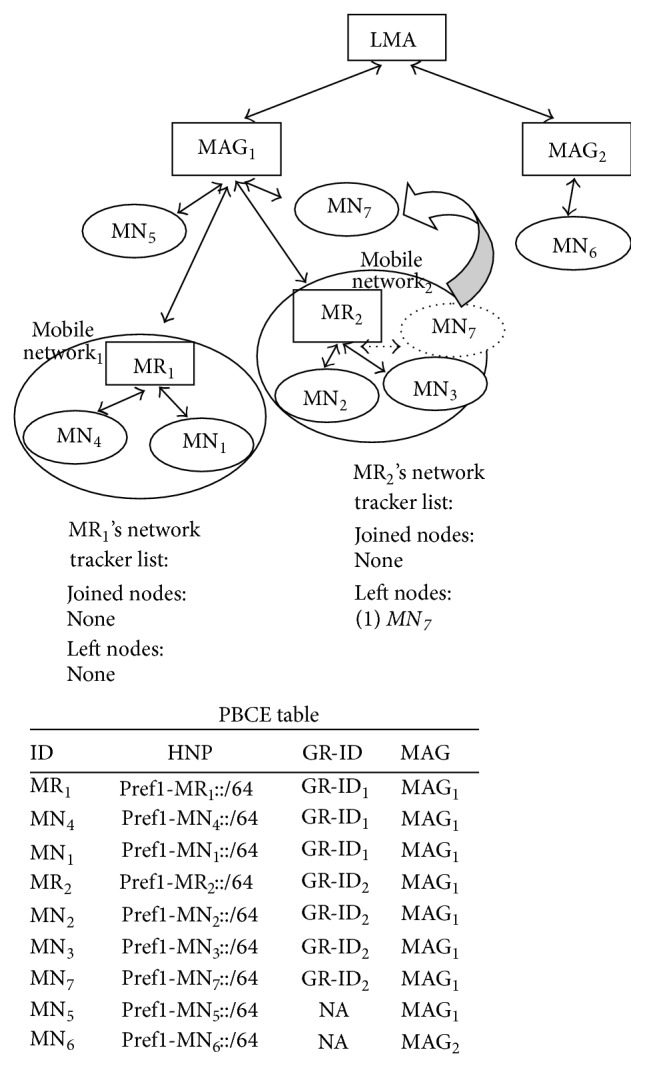
Intra-MAG movement: mobile node detaches from mobile network and attaches to MAG.

*(a) Mobile Node Detaches from Mobile Network and Attaches to MAG*.  Figure 2 explains this scenario where mobile node detaches from mobile network and attaches to the MAG. MN_7_ which is part of Mobile Network_2_ detaches from MR_2_ and attaches to MAG_1_. MR_2_ updates its tracker list. MN_7_ is still under the same MAG. So MAG does not send PBU for this mobile node movement to LMA. So PBCE remains same.

**Figure 3:**
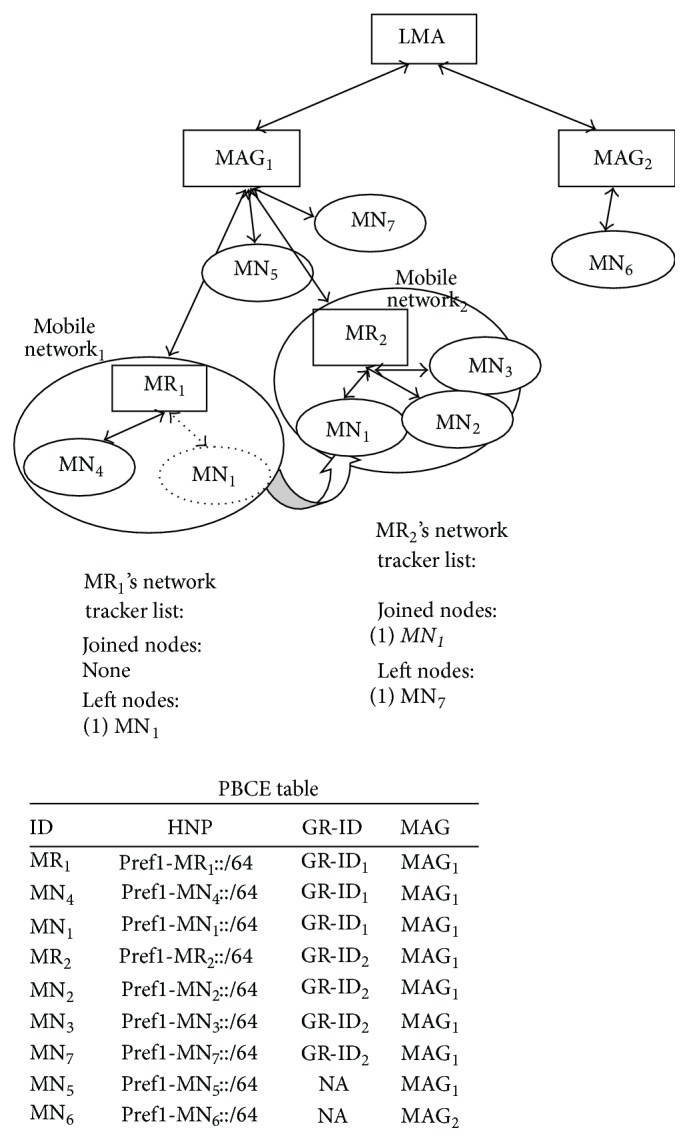
Intra-MAG movement: mobile node moves between mobile networks which are under the same MAG.

*(b) Mobile Node Moves between Mobile Networks Which Are under the Same MAG*.  Figure 3 explains the scenario, where a mobile node moves between mobile networks which are under the same MAG. MN_1_ which is part of Mobile Network_1_ detaches from MR_1_, attaches to MR_2_ and becomes part of Mobile Network_2_. MR_1_ and MR_2_ update their network tracker list. MN_7_ is still under the same MAG. So in this situation also, MAG does not inform about the mobile node movement to LMA. So PBCE remains the same.

**Figure 4:**
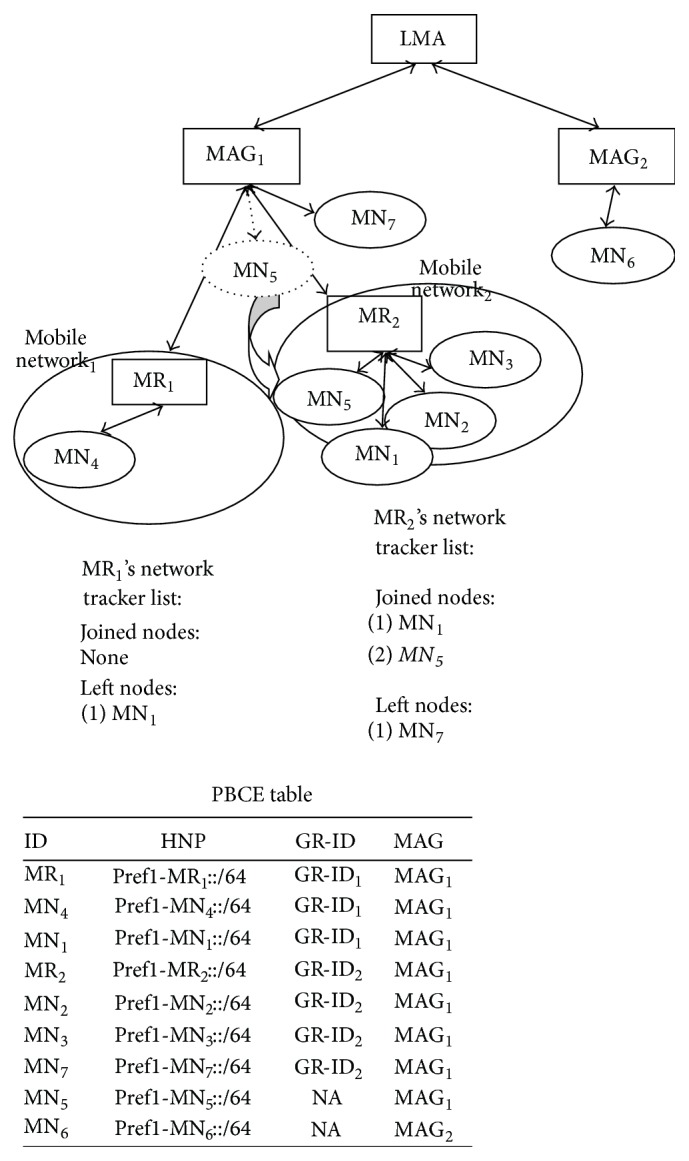
Intra-MAG movement: mobile node detaches from MAG and attaches to mobile network.

*(c) Mobile Node Detaches from MAG and Attaches to Mobile Network*.  Figure 4 explains the scenario, where a mobile node detaches from MAG and attaches to a mobile network. MN_5_ detaches from MAG_1_ and attaches to Mobile Network_2_. MR_2_ updates its network tracker list with the joined node. MN_5_ is still under the same MAG. So LMA is not informed about the mobile network movement. So PBCE remains same.

#### 3.2.2. Inter-MAG Movement

**Figure 5:**
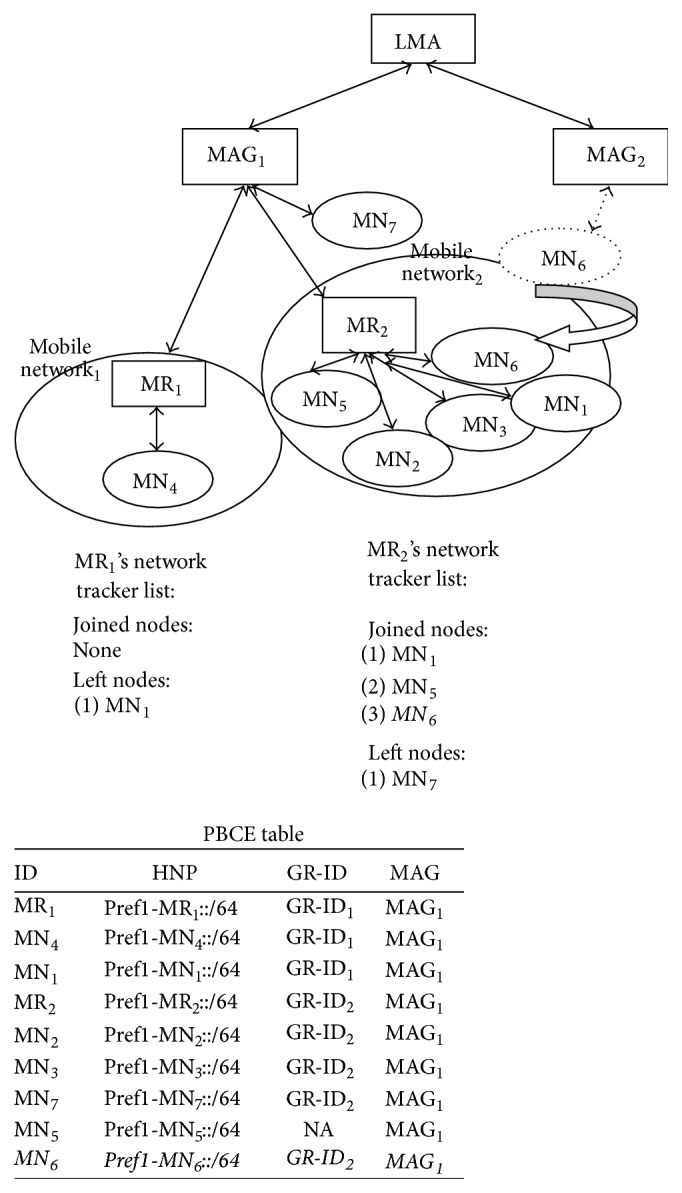
Inter-MAG movement.

Figure 5 explains the scenario, where mobile node moves between MAGs. MN_6_ detaches from MAG_2_ and attaches to Mobile Network_2_. MR_2_ updates its tracker list with the added node. MN_6_ is the new node that joined under MAG. So PBU and PBA are exchanged and MAG's PBUL and LMA's PBCE table are updated.

### 3.3. Procedure When Mobile Network Changes Its Access Point

**Figure 6:**
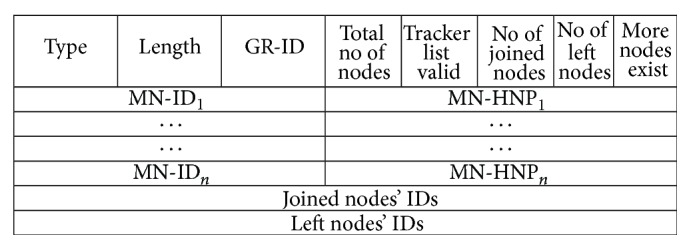
Mobile_Network_Location_Update (MNLU) message.

While mobile network changes its access point, the procedures to be followed to register mobile network's current location with LMA are given below:

1. Register mobile router with LMA.
2. Mobile router sees that it gets the same MNP and infers that it roams inside the same PMIPV6 network. So it sends information about the mobile network in the newly introduced Mobile_Network_Location_Update (MNLU) message, which is shown in Figure 6. MNLU has two parts: (i) ID and MNP of mobile nodes in mobile network and (ii) mobile tracker list. Mobile router adds all the mobile network nodes and their MNPs to MNLU. Mobile tracker list is added to MNLU, only if the number of nodes in mobile tracker list is lesser than the number of nodes in mobile network. Also flag “Tracker list valid” is set to value “1” which indicates that MNLU has mobile tracker list.
3. The MAG strips off the MNN information and their MNPs. If flag “Tracker list” is “1,” then it sends mobile tracker list to LMA. Otherwise, it adds only IDs of all mobile nodes in MNLU and sends it to LMA.
4. If flag “More Nodes Exist” is set to “1,” it indicates that more nodes exist in mobile network apart from the nodes given in MNLU. If flag “More Nodes Exist” is set to “0,” it indicates that no more nodes are present in mobile network.
5. On receiving MNLU, LMA updates PBCE as per the below rules:
  - If flag “Tracker list” is “0,” it means that MNLU has IDs of all the nodes in mobile network. LMU updates the respective PBCE entries with the new MAG. PBCE entries of the nodes, which were part of mobile network, but not present in MNLU, are still holding the same GR-ID. LMU deletes GR-ID from those entries and retains the same MAG.
  - If flag “Tracker list” is “1,” it means that MNLU has only the mobile tracker list. LMU updates PBCE using “Joined nodes” list and “Left nodes” list:
    - The nodes which are present in “Joined nodes” list: GR-ID is updated with its new mobile network's Group ID. MAG information is updated with which the mobile network is currently attached.
    - The nodes which are present in “left nodes” list: if PBCE has the same GR-ID, then GR-ID is deleted and MAG information is retained.
6. Mobile router resets its mobile tracker list and starts tracking the MNN movements freshly.

**Figure 7:**
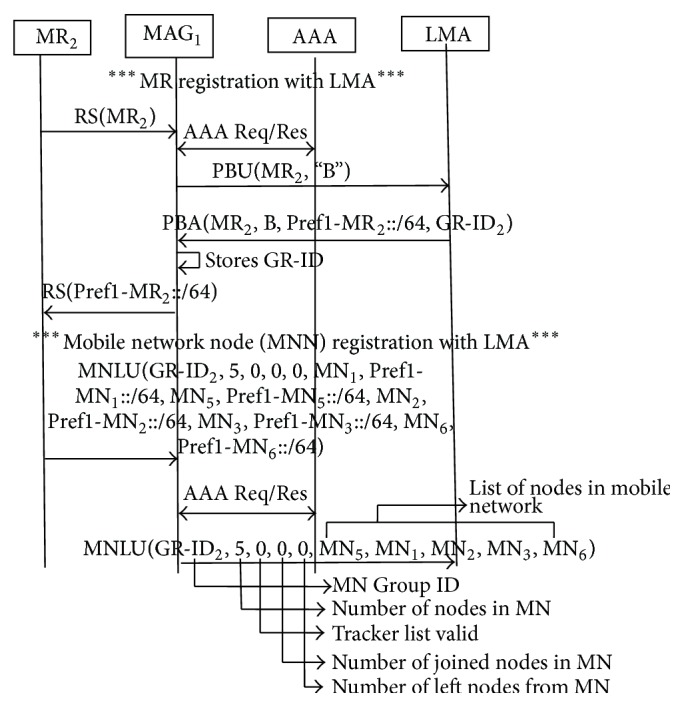
Message flow while mobile network changes its MAG.

**Figure 8:**
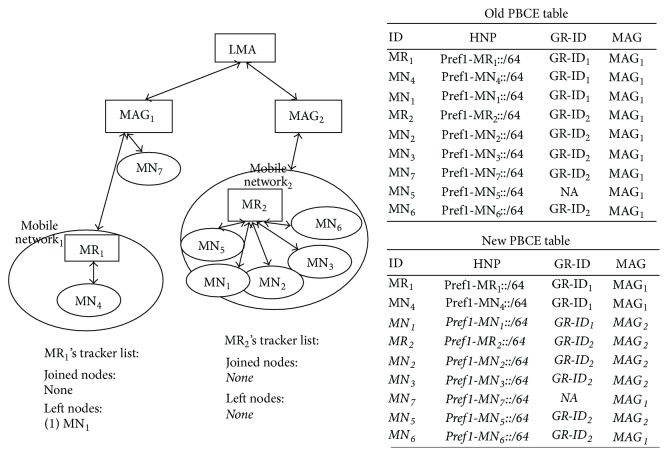
Updated PBCE and mobile network's new tracker list.

Using the above procedure, MAG and LMA are in sync on the mobile network information. Then, they can start transmission of mobile network's packets.  Figure 7 shows the message flow, while mobile network attaches to MAG_2_. Initially, MR_2_ is registered with LMA. Then, MR sends MNLU message to MAG. It does not send tracker list in MNLU, as the number of nodes in mobile tracker list is equal to the number of nodes in mobile network. MAG strips off the MN-IDs and their MNP from MNLU. It adds only the MN-IDs of nodes in mobile network and forwards it to LMA.  Figure 8 shows the mobile network's inter-MAG movement and the changes made in PBCE and MR_2_'s mobile network tracker list. PBCE entries of the nodes which are currently part of mobile network are updated with the Group ID and new MAG ID. Group ID is removed from the PBCE entries of the nodes which are no longer a part of mobile network. MR_2_ resets its network tracker list to start mobile node's tracking afresh.

The proposed architecture groups the mobile network nodes and assigns unique Group ID to it. During mobile network movement, mobile network's new location is updated to LMA using very little information (Group ID and delta network changes). This architecture uses very little information to update mobile network location compared to N-NEMO and it does not need frequent PBU to maintain mobile networks' group information as needed in N-PMIPV6. It combines the advantages of both N-NEMO and N-PMIPV6.

## 4. Performance Evaluation

The proposed architecture's enhancement is realized in terms of load at LMA, number and size of mobility signaling messages to be exchanged to update change in group members of a mobile network, and handover time. Performance of the proposed architecture is compared against N-NEMO and N-PMIPV6. Acronyms used in performance evaluation are given as follows:

 
*N*_PBU_: number of PBUs exchanged,
 
*N*_PBA_: number of PBAs exchanged,
 
*N*_join_IntraMAG_, *N*_join_InterMAG_: number of nodes joined in mobile network due to intra- and inter-MAG movement, respectively,
 
*N*_join_, *N*_left_: number of nodes that joined or left mobile network, respectively,
 
*S*_PBU_: total size of PBU exchanged,
 
*S*_PBA_: total size of PBA exchanged,
 SC: total signaling cost,
 LMA_load_: load at LMA,
 
*N*_mobile_network_: number of nodes in mobile network,
 Bytes_node_: number of bytes to specify the mobile node information,
 Bytes_MNP_: number of bytes to specify mobile nodes MNP,
 
*S*_PBU_PMIPV6_: size of PBU described in basic PMIPV6,
 
*S*_PBA_PMIPV6_: size of PBA described in basic PMIPV6.

### 4.1. Number of Mobility Signaling Messages Exchanged during MNN/Mobile Network Movement

During MNN/mobile network movement, PBU and PBA should be exchanged between MAG and LMA to inform new location of MNN/mobile network. The number of PBUs and PBAs varies depending on the architecture and is given in Table 1. In N-PMIPV6, PBU and PBA are exchanged during mobile node's intra-MAG movement (i.e., movement between mobile networks which are connected to the same MAG and movement from MAG to mobile network and vice versa) and inter-MAG movement. But N-NEMO and the proposed architecture defer sending PBU and PBA during intra-MAG movement.

**Figure 9:**
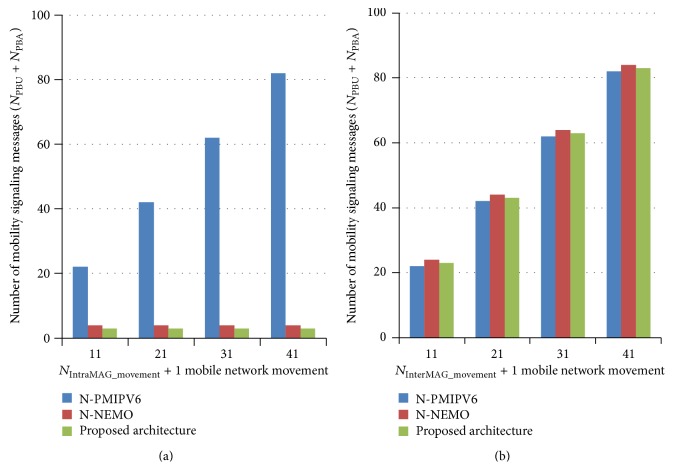
(a) *N*_PBU_ + *N*_PBA_ by varying *N*_IntraMAG_movement_. (b) *N*_PBU_ + *N*_PBA_ by varying *N*_InterMAG_movement_.

Figure 9(a) shows the number of PBUs and PBAs exchanged in all three architectures with varying *N*_InterMAG_movement_ and a mobile network movement. It shows that the proposed architecture and N-NEMO exchange lessen mobility signaling, as they are exchanged only during mobile network movement, not during mobile node's intra-MAG movement. In N-PMIPV6, the number of mobility signaling increases, as the number of mobile node's intra-MAG movements increases. In Figure 9(b), the number of PBUs and PBAs exchanged in all three architectures with varying *N*_InterMAG_movement_ and a mobile network movement are shown. All three architectures show similar performance as all of them exchange mobility signaling during inter-MAG movement and mobile network movement.

### 4.2. Size of Mobility Signaling Messages during Mobile Network Movement

All three architectures use the same mobility signaling structure during MNN movement. So only the size of mobility signaling messages exchanged during mobile network movement is taken for performance analysis. It differs based on different architectures as given in Table 2. N-PMIPV6 and N-NEMO have not clearly defined how they get mobile node's MNP from LMA. This performance evaluation uses the mechanism defined by Jeon et al. [14] to get MNP from LMA during mobile network movement for N-NEMO and N-PMIPV6 architectures. In the existing architectures, increase in the size of PBU and PBA is proportional to the number of nodes in mobile network. In the proposed architecture, size of PBU depends on the number of nodes in network tracker list (number of nodes joining in and leaving mobile network). Also the size of PBA is always the same irrespective of the number nodes in mobile network or membership changes in mobile network. So size of mobility signaling in the proposed architecture is always lesser than the existing architectures.

### 4.3. Total Signaling Cost Computed with MNN and Mobile Network Movement

Signaling cost is measured by adding total size of PBU and PBA exchanged between mobile MAG and LMA:

(1) $$\begin{array}{c} \text{Total signaling cost (SC)}=\text{Cost of PBU}+\text{Cost of PBA}=\left(N_{\text{PBU}}*S_{\text{PBU}}\right)+\left(N_{\text{PBA}}*S_{\text{PBA}}\right). \end{array}$$

(a) In N-PMIPV6:
 In N-PMIPV6, signaling cost is measured as below:
  (2) $$\begin{array}{c} \text{SC}=\left(N_{\text{IntraMAG\_movement}}+N_{\text{InterMAG\_movement}}+1\right)*S_{\text{PBU\_PMIPV6}}+\left(N_{\text{IntraMAG\_movement}}+N_{\text{InterMAG\_movement}}+1\right)*S_{\text{PBA\_PMIPV6}}+\left(N_{\text{mobile\_network}}*\left({\text{Bytes}}_{\text{node}}+{\text{Bytes}}_{\text{MNP}}\right)\right)=\left(N_{\text{IntraMAG\_movement}}+N_{\text{InterMAG\_movement}}+1\right)*\left(S_{\text{PBU\_PMIPV6}}+S_{\text{PBA\_PMIPV6}}\right)+\left(N_{\text{mobile\_network}}*\left({\text{Bytes}}_{\text{node}}+{\text{Bytes}}_{\text{MNP}}\right)\right). \end{array}$$
(b) In N-NEMO:
 In N-NEMO, signaling cost is measured as below:
  (3) $$\begin{array}{c} \text{SC}=\left(N_{\text{InterMAG\_movement}}+1\right)*\left(S_{\text{PBU\_PMIPV6}}\right)+\left(N_{\text{InterMAG\_movement}}+1\right)*S_{\text{PBA\_PMIPV6}}+\left(N_{\text{mobile\_network}}*{\text{Bytes}}_{\text{node}}\right)+\left(N_{\text{mobile\_network}}*{\text{Bytes}}_{\text{MNP}}\right)=\left(N_{\text{InterMAG\_movement}}+1\right)*\left(S_{\text{PBA\_PMIPV6}}+S_{\text{PBU\_PMIPV6}}\right)+\left(N_{\text{mobile\_network}}\right)\left({\text{Bytes}}_{\text{node}}+{\text{Bytes}}_{\text{MNP}}\right). \end{array}$$
(c) In Proposed Architecture,
  (4) $$\begin{array}{c} \text{SC}=\left(N_{\text{InterMAG\_movement}}+1\right)*\left(S_{\text{PBU\_PMIPV6}}\right)+\left(N_{\text{InterMAG\_movement}}*S_{\text{PBA\_PMIPV6}}\right)+S_{\text{MNLU}}+\left(X*{\text{Bytes}}_{\text{node}}\right)=\left(N_{\text{InterMAG\_movement}}*\left(S_{\text{PBA\_PMIPV6}}+S_{\text{PBU\_PMIPV6}}\right)\right)+S_{\text{PBU\_PMIPV6}}+S_{\text{MNLU}}+\left(X*{\text{Bytes}}_{\text{node}}\right), \end{array}$$
 where
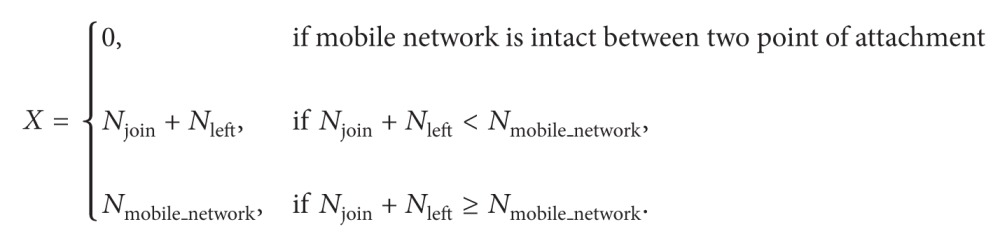
(5)

Equation (2) indicates that signaling cost of N-PMIPV6 increases as the number of nodes joining or leaving the mobile network increases. Equation (3) indicates that, in N-NEMO, information of all nodes in mobile network is exchanged irrespective of change in mobile network. Equations (4) indicates that, in the proposed architecture, only delta network changes are exchanged unless delta network changes grow bigger than the number of nodes in mobile network. From (2), (3), and (4), it is evident that total signaling cost is always lesser than N-PMIPV6 and N-NEMO except for one scenario, where the number of nodes in tracker list is greater than the number of nodes in mobile network. In the exceptional scenario also, the proposed architecture matches the performance analysis of N-NEMO.

The performance analysis on signaling cost is done for all three architectures in various mobile network conditions as described below. The parameters used in the performance evaluation are of variable size parameters. By using the minimum required size, the following values are used for those parameters throughout the simulation *S*_PBU_PMIPV6_ = *S*_PBA_PMIPV6_ = 76 bytes, *S*_MNLU_ = 44 Bytes (length of IPV6 header + part of MNLU without mobile node's info), and Bytes_MNP_ = Bytes_node_ = 4 bytes.

#### 4.3.1. Analysis on Signaling Cost While Mobile Network Changes Its Access Point with Varying Number of Nodes in Mobile Network

**Figure 10:**
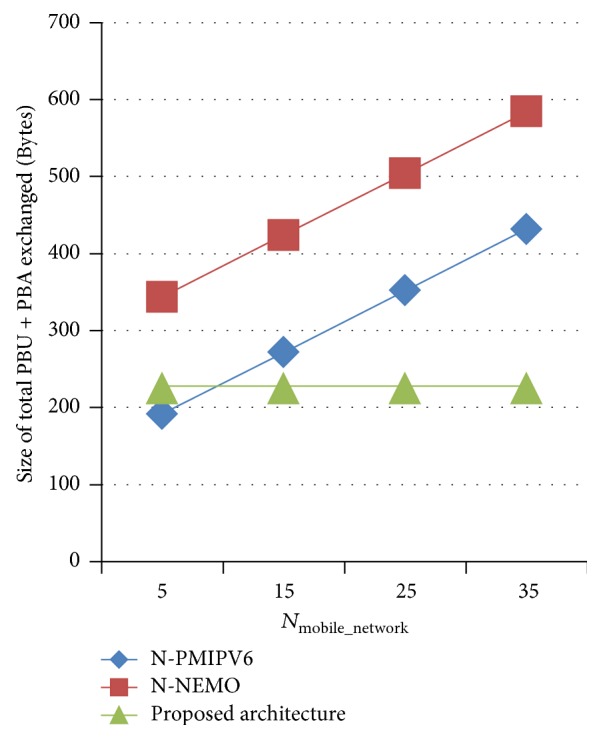
Analysis on signaling cost while varying *N*_mobile_network_.

The performance analysis is done on signaling cost while mobile network changes its access point with varying number of nodes in mobile network. Simulation is done in such a way that mobile network is intact between different access points (no joined nodes/no left nodes) and size of PBU and PBA exchanged is measured.  Figure 10 shows the analysis result. The proposed architecture sends only delta network changes to LMA. As network is intact between two access points, size of mobility signaling of the proposed architecture is constant irrespective of the number of nodes in mobile network and lesser compared to N-NEMO and N-PMIPV6. In N-NEMO and N-PMIPV6, the size of signaling messages increases as the number of nodes in mobile network increases.

#### 4.3.2. Analysis on Signaling Cost with Varying Number of Mobile Node's Intra-MAG Movement Followed by a Mobile Network Movement

**Figure 11:**
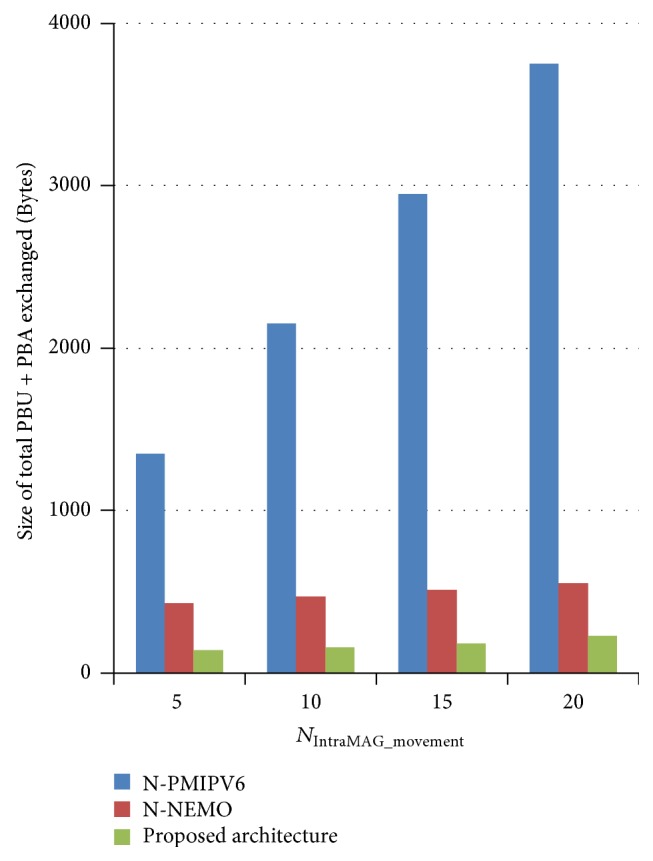
Analysis on signaling cost while varying *N*_joined_.

Impact of mobile node's inter-MAG movement is the same in all three architectures. So the signaling cost is measured only by varying mobile node's intra-MAG movement followed by a mobile network movement. Initially, mobile network has 50 MNNs and simulation is conducted by adding new mobile nodes followed by a mobile network movement. Simulation results are shown in Figure 11. In N-PMIPV6, as PBU and PBA are exchanged even for single mobile node mobility in and out of mobile network, size of signaling messages is proportional to the number of nodes joined. N-NEMO and the proposed architecture exchange mobility signaling only while the mobile network changes its access point. So they exhibit lesser size of mobility signaling to be processed. Also the proposed architecture sends only the delta network changes. So it exhibits even lesser size of mobility signaling messages compared to N-NEMO.

#### 4.3.3. Analysis of Signaling Cost after Introducing Random Mobility to the Mobile Nodes

**Figure 12:**
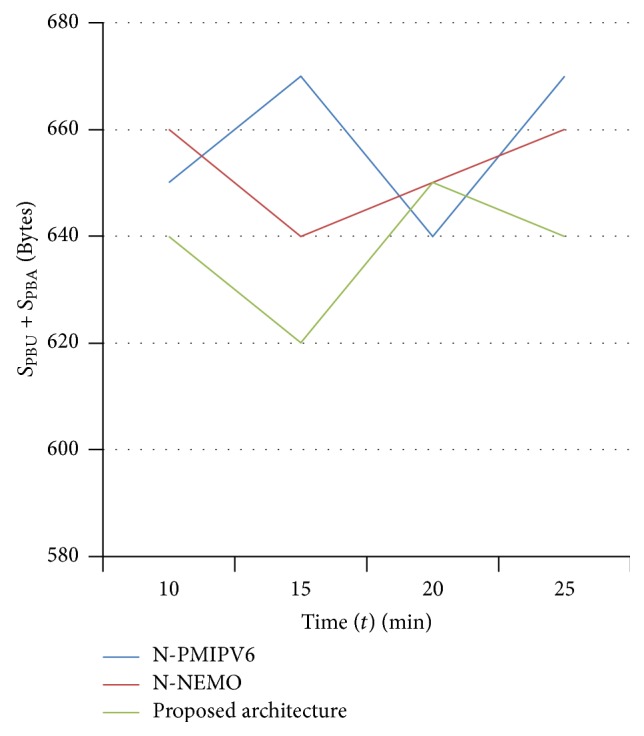
Analysis on signaling cost by introducing random mobility to the mobile nodes.

The performance analysis is done by introducing the random mobility to the mobile nodes and mobile network and the result is shown in Figure 12. In specific time interval, the sizes of PBU and PBA to be exchanged are measured for all three architectures.

### 4.4. Load at LMA

Load at LMA is defined as consolidation of processing cost of PBU and processing cost of PBA as described below:

(6) $$\begin{array}{c} {\text{LMA}}_{\text{load}}=\text{Cost of PBU}+\text{Cost of PBA}. \end{array}$$

As LMA is the single point to send or receive packets from outside of PMIPV6 network, it is very much important to reduce the load on LMA. As described above in previous sections, total size of mobility signaling to be processed in the proposed architecture is always lesser than other architectures except while delta network changes exceed the number of nodes in mobile network.

### 4.5. Advantages and Disadvantages of the Proposed Architecture

Existing architectures N-NEMO and N-PMIPV6 are considered to be efficient architectures to support NEMO in PMIPV6. N-NEMO outperforms N-PMIPV6, if the movement of mobile nodes in mobile network is less. N-PMIPV6 exhibits better performance, if the movement of mobile nodes in mobile network is huge. The proposed architecture possesses the goodness of both of the existing architectures. Advantages and disadvantages of the proposed architecture are discussed below.

Advantages are as follows:

1. The proposed architecture completely eliminates the exchange of mobility signaling while mobile node changes its access point under the same MAG, that is, movement between mobile networks which are connected to same MAG and movement from MAG to mobile network and vice versa.
2. If the number of entries in the mobile tracker list is lesser (i.e., the movement of mobile nodes in mobile network is less) than the number of nodes in mobile network, then the proposed architecture matches the performance of N-PMIPV6 and outperforms N-NEMO.
3. If the number of entries in the mobile tracker list exceeds (i.e., the movement of mobile nodes in mobile network is huge) the number of nodes in mobile network, then the proposed architecture matches the performance of N-NEMO and outperforms N-PMIPV6.
4. The proposed architecture does not exhibit poorer performance than existing architectures in any scenario.

Disadvantages are as follows:

1. The proposed architecture requires changes in mobile router also. But this is unavoidable to ensure IP session continuity while a mobile node moves between PMIPV6 network and mobile network which is attached to the same PMIPV6 network. All the architectures [7–11, 14], which try to add NEMO in PMIPV6 and ensure IP session continuity, propose changes in mobile router.

### 4.6. Comparative Study between N-NEMO, N-PMIPV6, and Proposed Architecture

Comparative study between N-NEMO, N-PMIPV6, and proposed architecture is given in Table 3.

## 5. Conclusions

PMIPV6 protocol supports mobile node's seamless mobility in PMIPV6 network without the mobile node involvement. This research work enhances PMIPV6 to support mobile network mobility. It defers sending mobility signaling to LMA as long as the mobile node is roaming and attaching to different mobile routers which are under the same MAG. While mobile network moves across access points, only delta network changes are sent to LMA, instead of sending complete network information. So the proposed architecture requires lesser mobility signaling to be exchanged compared to other architectures which support network mobility in PMIPV6 network. Lesser mobility signaling ensures lesser burden in LMA which is a crucial point to route packets in and out of mobile network. The performance analysis proves that the proposed architecture shows better results compared to N-NEMO and N-PMIPV6 during mobile node's intra-MAG movement and mobile network movement.

## Figures and Tables

**Table 1:** *N*_PBU_ and *N*_PBA_ exchanged during MNN/mobile network movement.

	*N*_PBU_		*N*_PBA_	
	During MNN movement	During mobile network movement	During MNN movement	During mobile network movement
N-PMIPV6	*N*_IntraMAG_movement_ + *N*_InterMAG_movement_	1	*N*_IntraMAG_movement_ + *N*_InterMAG_movement_	1

N-NEMO	*N*_InterMAG_movement_	2 (1 for MR + 1 for mobile nodes in mobile network)	*N*_InterMAG_movement_	2 (1 for MR + 1 for mobile nodes in mobile network)

Proposed architecture	*N*_InterMAG_movement_	2 (1 for MR + 1 for mobile nodes in mobile network)	*N*_InterMAG_movement_	1 (1 for MR)

**Table 2:** Size of PBU and PBA exchanged while mobile network changes its access point.

	To register MR		To register nodes in mobile network	
	*S*_PBU_	*S*_PBA_	*S*_PBU_	*S*_PBA_
N-PMIPV6	*S*_PBU_PMIPV6_	*S*_PBA_PMIPV6_ + (*N*_mobile_network_ *∗*(Bytes_node_ + Bytes_MNP_))	×	×

N-NEMO	*S*_PBU_PMIPV6_	*S*_PBA_PMIPV6_	*S*_PBU_PMIPV6_ + (*N*_mobile_network_ *∗*Bytes_node_)	*S*_PBA_PMIPV6_ + (*N*_mobile_network_ *∗*Bytes_MNP_)

Proposed architecture	*S*_PBU_PMIPV6_	*S*_PBA_PMIPV6_	*S*_MNLU_ + (*X∗*Bytes_node_) where *X* =	×
			{	
			$\begin{array}{cc} \;0, & {\;\;\;\;\;\;\;N}_{\text{join}}+N_{\text{left}}=0 \end{array}$	
			$\begin{array}{cc} {\;N}_{\text{join}}+N_{\text{left}}, & \;{\;\;N}_{\text{join}}+N_{\text{left}}<N_{\text{mobile\_network}} \end{array}$	
			$\begin{array}{cc} \;N_{\text{mobile\_network}}, & N_{\text{join}}+N_{\text{left}}\geq N_{\text{mobile\_network}} \end{array}$	
			}	

**Table 3:** Comparative study.

	N-PMIPV6	N-NEMO	Proposed architecture
Nodes in mobile nodes grouped	Yes	Yes	Yes

Mobility signaling exchanged during mobile node's inter-MAG movement	Yes	Yes	Yes

Mobility signaling exchanged during mobile node's intra-MAG movement	Yes	No	No

Amount of information shared during mobile network movement	Information of all the nodes in mobile network is shared	Information of all the nodes in mobile network is shared	Only delta network information is shared
